# The *Staphylococcus epidermidis* Transcriptional Profile During Carriage

**DOI:** 10.3389/fmicb.2022.896311

**Published:** 2022-04-26

**Authors:** Pascâl Teichmann, Anna Both, Christiane Wolz, Mathias W. Hornef, Holger Rohde, Amir S. Yazdi, Marc Burian

**Affiliations:** ^1^Department of Dermatology and Allergology, RWTH University Hospital Aachen, Aachen, Germany; ^2^Institute of Medical Microbiology, Virology and Hygiene, University Hospital Hamburg-Eppendorf, Hamburg, Germany; ^3^Interfaculty Institute of Microbiology and Infection Medicine, University of Tuebingen, Tuebingen, Germany; ^4^Institute of Medical Microbiology, RWTH University Hospital Aachen, Aachen, Germany

**Keywords:** *in vivo* gene expression, human colonization, nasal colonization, skin colonization, global regulators, bacterial adaptation, staphylococcal accessory regulatorA (*sar*A)

## Abstract

The virulence factors of the opportunistic human pathogen *Staphylococcus epidermidis* have been a main subject of research. In contrast, limited information is available on the mechanisms that allow the bacterium to accommodate to the conditions during carriage, a prerequisite for pathogenicity. Here, we tested the hypothesis that the adaptation of *S. epidermidis* at different anatomical sites is reflected by differential gene regulation. We used qPCR to profile *S. epidermidis* gene expression *in vivo* in nose and skin swabs of 11 healthy individuals. Despite some heterogeneity between individuals, significant site-specific differences were detected. For example, expression of the *S. epidermidis* regulator *sar*A was found similarly in the nose and on the skin of all individuals. Also, genes encoding colonization and immune evasion factors (*sdr*G, *cap*C, and *dlt*A), as well as the sphingomyelinase encoding gene *sph*, were expressed at both anatomical sites. In contrast, expression of the global regulator *agr* was almost inactive in the nose but readily present on the skin. A similar site-specific expression profile was also identified for the putative chitinase-encoding SE0760. In contrast, expression of the autolysine-encoding gene *sce*D and the wall teichoic acid (WTA) biosynthesis gene *tag*B were more pronounced in the nose as compared to the skin. In summary, our analysis identifies site-specific gene expression patterns of *S. epidermidis* during colonization. In addition, the observed expression signature was significantly different from growth *in vitro*. Interestingly, the strong transcription of sphingomyelinase together with the low expression of genes encoding the tricarboxylic acid cycle (TCA) suggests very good nutrient supply in both anatomical niches, even on the skin where one might have suspected a rather lower nutrient supply compared to the nose.

## Introduction

The human nose is an important colonization site for two major staphylococcal species, *Staphylococcus* (*S*.) *aureus* and *Staphylococcus epidermidis*, the latter also being a ubiquitous colonizer of healthy skin (Wertheim et al., 2005; Du et al., 2021). While *S. epidermidis* is primarily considered as a commensal bacterium with beneficial properties, for example, in modulating the immune system (Naik et al., 2012) and protecting against colonization by pathogens (Cogen et al., 2010), it also possesses selective pathogenic potential to cause nosocomial infections associated with implanted medical devices (Harris et al., 2010; Tande and Patel, 2014).

The pathogenic lifestyle of this opportunistic pathogen has been the main subject of research, and its ability to form biofilm is recognized as a major virulence factor (Otto, 2009, 2014; Buttner et al., 2015). In contrast, there is limited information on how the bacterium adapts during colonization and how environmental factors influence its commensal lifestyle. In particular, the skin at various anatomical sites with differences in temperature, pH, moisture, and sebum content provides strongly contrasting microenvironments (Grice et al., 2009; Grice and Segre, 2011). The different properties of the skin sites influence the composition of the local microbial community, which varies depending on the skin region. For example, Staphylococci and Corynebacteria preferentially colonize moist areas of the skin (Byrd et al., 2018). Despite varying external influences, the composition of the skin’s microbial communities in one individual is largely stable over time (Oh et al., 2016).

The fact that *S. epidermidis*, as one of the most abundant bacterial species on the skin (Byrd et al., 2018), can act as either pathogen or commensal suggests dynamic and tunable virulence gene regulation. Gene regulation is facilitated by global regulators that are part of a complex network that integrates information such as cell density, nutritional, and other environmental signals, or stress such as exerted by antimicrobial peptides (AMPs) (Vuong et al., 2005; Li et al., 2007; Otto, 2009). Besides transcriptional regulation, the transition of *S. epidermidis*, from the commensal to infectious phenotype, also occurs through genomic changes. Both et al. (2021) demonstrated that the crucial gene loci can almost exclusively be assigned to the mobilome, which is defined as a group of exchangeable elements such as phage-encoded genes or mobile genetic elements. The adaptation to the infectious lifestyle is characterized by increased biofilm formation and enhanced growth on nutrient-poor media, as well as a reduced production of hemolysins (Both et al., 2021). The relevance of *S. epidermidis* gene regulation is illustrated by the fact that expression and secreting the protease EcpA aggravate the clinical symptoms in patients with atopic dermatitis (Cau et al., 2021). Thus, the bacterium acts context-dependent and not always beneficial. Recently, we could show that the integrity of the skin barrier determines whether *S. epidermidis* colonizes the skin as friend or foe. When the skin barrier is disrupted, *S. epidermidis* loses its protective function and its presence leads to increased colonization with *S. aureus* (Burian et al., 2017).

To gain insights into the commensal lifestyle of *S. epidermidis* and identify relevant determinants during colonization, we performed direct transcript analysis in the authentic human environment, considering its two major ecological niches: the nose and the skin. Using our direct *in vivo* qPCR approach, we here reveal for the first time the niche-specific expression pattern of *S. epidermidis* during its commensal lifestyle.

## Materials and Methods

### Ethics Statement

Skin and nasal swabs were obtained from healthy volunteers. This approach was approved by the local ethics committee of the Medical Faculty RWTH University of Aachen, Germany (EK 173/20). In accordance with the Declaration of Helsinki Principles guidelines, written consent was obtained from all participants involved in the study.

### Study Population, Sampling, and Growth of Swab Material

Eleven healthy volunteers [nine females and two males, median age 31.4 years (range 22–44 years)] were included in this study. Volunteers with medical history of systemic diseases, such as autoimmune diseases, diabetes mellitus, and cancer as well as chronic skin diseases (eczema or psoriasis), immune deficiency, or recent antibiotic use, were excluded. Concurrent colonization with *Staphylococcus aureus* was also an exclusion criterion.

For *in vivo* transcript analysis, a cotton wool swab was moistened in 300 μl nuclease-free water and the left and right anterior nares of the human volunteers were swabbed. Skin swabs were obtained by rotating the swab in the area between the toes. The swab was vigorously vortexed and 10 μl of the suspension was used for bacteriological and quantitative analyses. The cotton wool was removed from the swab using sterile tweezers and both the suspension and the cotton wool were directly treated with 1 ml of TRIzol™ LS reagent (Thermo Fisher Scientific) containing 0.5 ml of zirconia/silica beads [0.1 mm in diameter (Carl Roth)].

For *in vitro* transcript analysis, the native swab material was grown overnight in TSB medium (Carl Roth), diluted to an initial optical density value at 600 nm (OD_600_) of 0.05 in fresh medium and grown with shaking (180 rpm) at 37°C to the exponential (OD_600_ = 0.5) and post-exponential (OD_600_ = 0.5 + 4 h) phase. Bacteria were harvested by centrifugation and dissolved in 1 ml TRIzol™ reagent containing 0.5 ml of zirconia/silica beads.

### RNA Isolation, Reverse Transcription, and Quantitative Real-Time PCR

Bacteria were lysed using a high-speed homogenizer [FastPrep-24™ 5G (MP Biomedical)] twice at 6,500 rpm for 20 s and RNA isolation was performed as described previously (Burian et al., 2010b). To eliminate contaminating DNA, each *in vivo* RNA sample was digested with 8 U of RNase-free DNase I (Roche), 2 μl 10× incubation buffer (Roche), and 16 U of RNasin ribonuclease inhibitor (Promega) for 30 min at room temperature. DNase treatment was carried out twice for each sample. DNase I treatment was stopped using DNase inactivation reagent (Thermo Fisher Scientific). DNase treatment of *in vitro* samples was carried out using 0.05 μg of total RNA with adjusted amounts of reagents for a final volume of 50 μl.

Around 1 μl of *in vitro* and 3 μl of *in vivo* RNA were transcribed into cDNA using Super Script III Reverse Transcriptase (Thermo Fisher Scientific) and 200 ng of random hexamer primers (Thermo Fisher Scientific). Reverse transcription was performed as described in the instructions of the Superscript manufacturer. Complementary DNAs were diluted 1:3 with nuclease-free water (Thermo Fisher Scientific) and frozen at −20°C using Eppendorf LoBind Tubes (Eppendorf) for prolonged storage.

Relative quantification of *S. epidermidis* transcripts by qPCR was performed as described previously (Burian et al., 2021). Briefly, qPCR was carried out using the 7300 Real-Time PCR instrument (Applied Biosystems) in combination with the KAPA SYBR® FAST qPCR Master Mix (2×) ABI Prism (Merck). Master mixes were prepared as following: 8 μl KAPA SYBR® FAST qPCR Master Mix (2×) ABI Prism, 8 μl nuclease-free water, and 1 μl of each primer (see Supplementary Table 1). Finally, 2 μl of cDNA was added per reaction. Relative quantities of transcripts were calculated by a standard curve for each gene generated using 6-fold serial dilution of a *S. epidermidis* ATCC 12228 and RP62A wild-type cDNA mixture.

### Data Visualization and Statistical Analysis

Figures depicting differential gene expression as the ratio between *in vivo* and minimum or maximum expression *in vitro* were visualized as heat map (generated by GraphPad Prism 9.0.2). For this purpose, the ratio was calculated based on either the expression value at OD = 0.5 or OD = 0.5 + 4 h, depending on the time point at which the gene reaches its maximum expression *in vitro*. Subsequently, the data were logarithmically transformed to base 2. Accordingly, the ratio of *in vivo* to minimal transcription *in vitro* was also calculated.

Statistical analysis was performed with the Prism 9.0.2 package (GraphPad Software) using the Kruskal-Wallis test. Value of *p* < 0.05 was considered to be statistically significant.

## Results

### *In vivo* Transcriptional Analysis During Human *Staphylococcus epidermidis* Colonization

**Figure 1:**
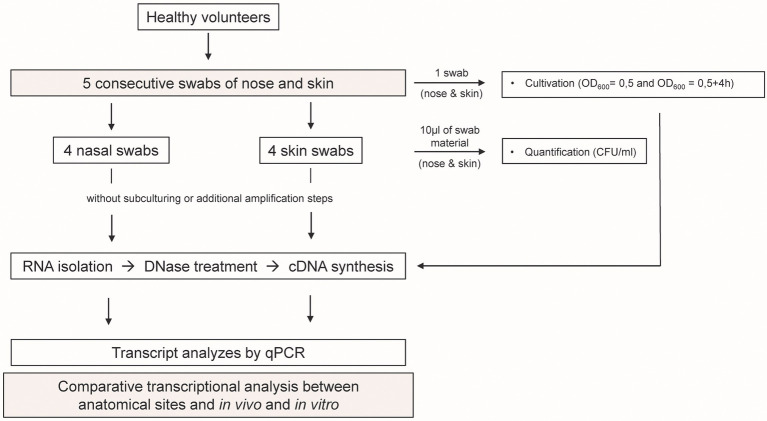
Study design. Five swabs from the nose and skin were obtained from 11 healthy individuals. RNA was isolated directly from the material of four swabs, and bacterial transcription was profiled using qPCR. In parallel, one swab from each individual was used for cultivation to exponential (OD_600_ = 0.5) and post-exponential (OD_600_ = 0.5 + 4 h) growth phase. RNA was isolated and bacterial transcription was measured by qPCR. *In vivo* gene expression in nose and skin was then compared with the *in vitro* expression pattern.

**Figure 2:**
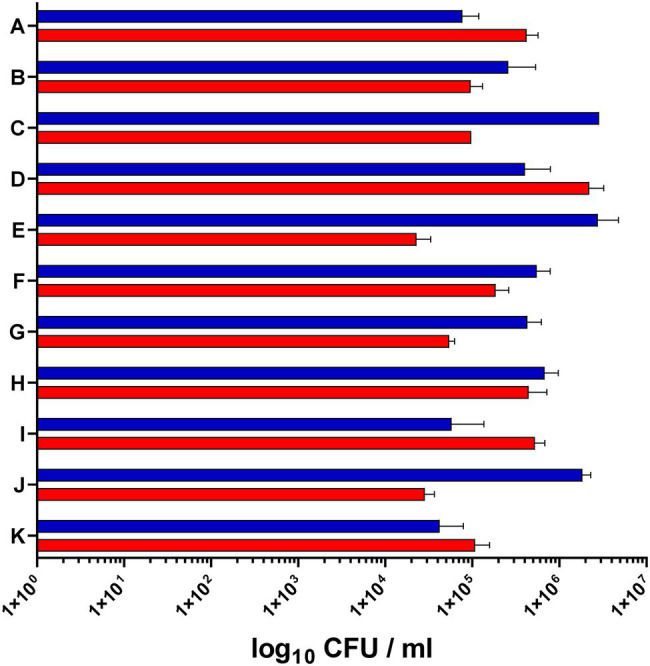
*Staphylococcus epidermidis* load in the nose and skin of 11 healthy volunteers. The bacterial load in the nose (red bars) and on the skin (blue bars) was determined by serial dilution and plating of swab material (CFU, colony forming units).

For the analysis of *S. epidermidis* gene expression during human colonization, we obtained nose and skin swabs of 11 healthy individuals who were positive for *S. epidermidis* at both anatomical sites (Figure 1). The *S. epidermidis* load determined by culture in nose and skin material varied markedly between individuals (range_nose_ 4.2 × 10^4^–2.20 × 10^6^ CFU/ml and range_skin_ 4.04 × 10^4^–2.86 × 10^6^ CFU/ml). No significant difference in abundance was observed between the two niches (mean nose 4.1 × 10^5^ CFU/ml and mean skin 9.05 × 10^5^ CFU/ml; Figure 2).

**Figure 3:**
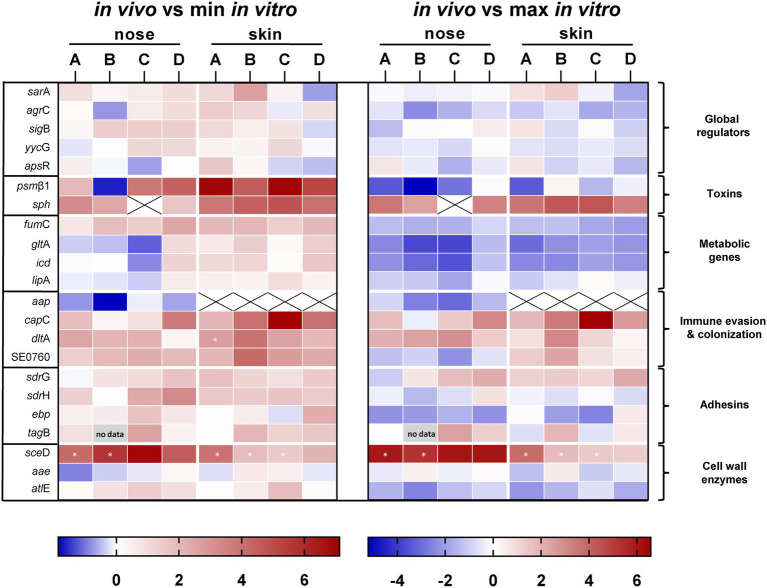
Direct transcript analysis of *Staphylococcus epidermidis* genes in the nose and skin of four healthy volunteers (A–D). Results are given as the ratio of transcription *in vivo* vs. minimal expression *in vitro* and vs. maximal expression *in vitro*. All data were log transformed (basis 2) and changes in gene expression were normalized in reference to the constitutively expressed gene *gyr*B. Genes colored red are those which were upregulated compared to *in vitro* and genes colored blue are those which were downregulated compared to *in vitro*. White cells indicate the same expression levels *in vivo* and *in vitro*. Results are the means of four separate samplings. White cells with an “x” have no values because gene expression was below the detection limit. Cells marked with an asterisk have no *in vitro* values because gene expression was below detection limit. For presentation reasons, the ratio was calculated from the mean value of the *in vitro* data of all subjects. The heat map was generated using GraphPad Prism 9.0.2.

**Figure 4:**
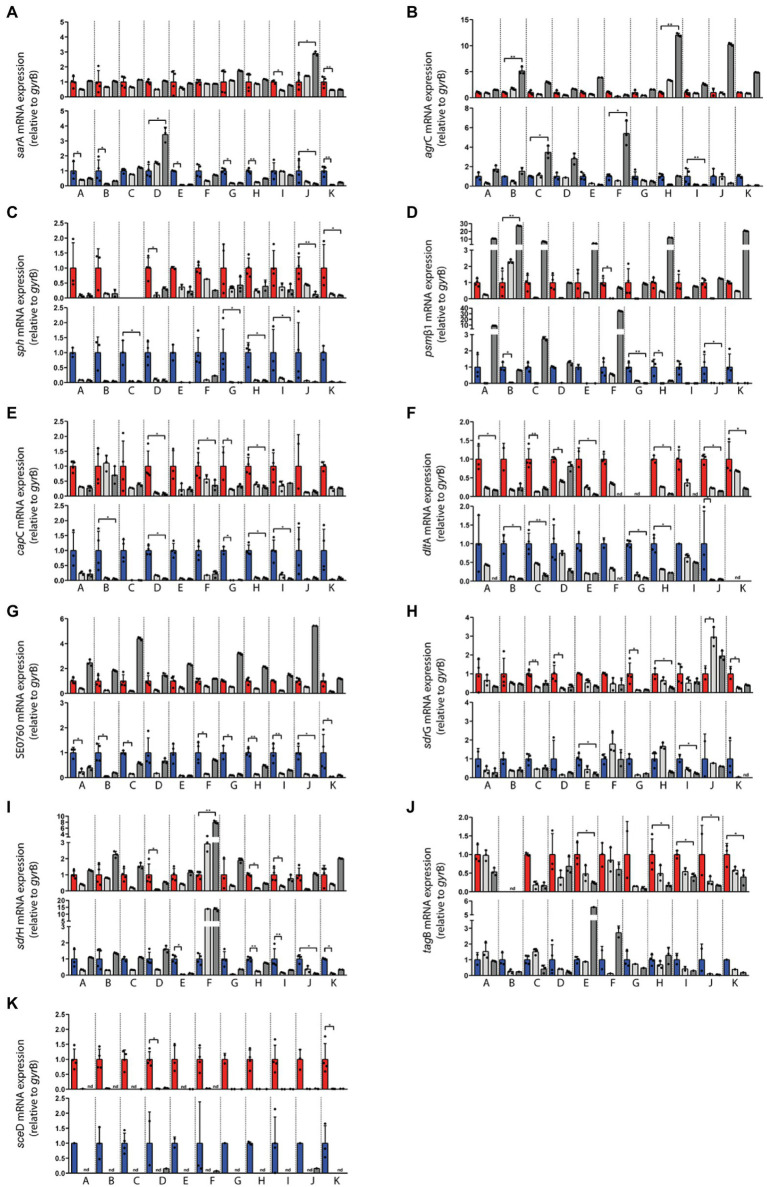
Transcriptional analysis of the indicated *Staphylococcus epidermidis* genes in swab material of the nose and skin of 11 healthy individuals **(A–K)**. Expression levels in nose swabs (upper panels, red columns) and skin swabs (lower panels, blue columns) as well as during exponential growth *in vitro* (light gray) and post-exponential growth *in vitro* (dark gray) were normalized to the expression level of the house keeping gene *gyr*B. Values from four independent samplings (four swabs) were used to calculate the mean expression *in vivo*. *In vitro* values represent technical replicates from one cultivation. The *in vitro* values were normalized to the respective *in vivo* level (set to 1). Statistically significant differences are indicated. ^*^p ≤ 0.05; ^**^p ≤ 0.01. Relative transcription of **(A)** *sar*A, **(B)** *agr*C, **(C)** *sph*, **(D)** *psm*β1, **(E)** *cap*C, **(F)** *dlt*A, **(G)** SE0760, **(H)** *sdr*G, **(I)** *sdr*H, **(J)** *tag*B, and **(K)** *sce*D. Gene name abbreviations, see Supplementary Table 2.

A total of five consecutive swabs were collected from each site, and four swabs were used directly for transcript analyses without sub-culturing or additional amplification steps (Figure 1). Initially, swabs were taken from four individuals and a total of 22 genes encoding bacterial factors involved in virulence regulation, toxin production, metabolism, adhesion, cell wall dynamics, immune evasion, and colonization were analyzed (see Supplementary Table 2). Results were visualized in a heat map as ratio between the expression *in vivo* and the minimum or maximum expression *in vitro* (Figure 3). No regulation or downregulation *in vivo* was detected for 11 genes. Therefore, we next focused on genes upregulated *in vivo* and genes with infection-associated biological significance. For these 11 remaining genes, swab samples from the four individuals mentioned above plus another seven individuals were analyzed. Results are shown as transcription *in vivo* (nose or skin) vs. transcription during growth *in vitro* (exponential and post-exponential growth phase) relative to the housekeeping gene *gyr*B (Figure 4). The main results can be functionally classified into the following groups.

#### Global Regulators

Expression of five global regulators *sar*A, *sig*B, *yyc*FG, *aps*XRS, and *agrC* of the *agr* quorum-sensing system was analyzed (Figure 3). *In vivo* transcriptional analyses of *sar*A revealed high expression in the nose, mostly within the range of maximal transcription *in vitro* (Figures 3, 4A). Moreover, *sar*A expression was even more pronounced in skin swabs, where it mostly exceeded the maximal *in vitro* levels (exception: individual C and D; Figure 4A), suggesting a general role of this regulatory protein during commensalism. In contrast to the high activity of *sar*A, the quorum-sensing regulator *agr* was predominantly not expressed during nasal colonization (Figures 3, 4B). On human skin, *agr* activity was very heterogeneous, as shown by the high transcription in six individuals (Figure 4B subjects E, G, H, I, J, K). A high individual heterogeneity was also observed for the alternative sigma factor B (*sig*B) and the two- and three-component regulatory systems *yyc*FG and *aps*XRS, respectively (Figure 3; Supplementary Figure 1).

#### Toxins

Lysis of host cells and bacterial spread especially during biofilm formation is mediated by a number of *S. epidermidis* toxins (Bresco et al., 2017). Similar to the heterogeneous *agr* expression on human skin, *psm*ß1 peptide was not uniformly expressed among individuals (Figures 3, 4D). While in the nose *psm*ß1 was strongly transcribed in five individuals, expression on the skin was upregulated in eight individuals (Figure 4D).

Interestingly, the sphingomyelinase encoding gene *sph* (also annotated as *hlb* in *S. aureus*) was highly transcribed in all individuals during both nasal and skin colonization. Only in the nose of subject C *sph* was not expressed (Figures 3, 4C).

#### Metabolic Genes

Similar to *S. aureus*, *S. epidermidis* TCA cycle activity is de-repressed during stationary phase *in vitro* (Wang et al., 2018). In the authentic human environment, we found the TCA encoding genes *fum*C, *glt*A, and *icd* expressed at low levels during asymptomatic colonization. Similarly, the lipase encoding gene *lip*A was weakly transcribed during nasal colonization. *lip*A expression on skin exhibits high individual heterogeneity (Figure 3; Supplementary Figure 1).

#### Immune Evasion and Colonization

In addition to its ability to form biofilm, *S. epidermidis* can produce a second exopolymer, poly-γ-glutamic acid (PGA), which is synthesized by the gene products of the *cap* locus (Kocianova et al., 2005). In our study, we investigated the transcription of *cap*C and *aap*, which is known to be involved in aggregation and biofilm formation (Hussain et al., 1997). While *aap* was only weakly transcribed and sometimes below the detection limit, *cap*C was strongly induced in both niches (Figures 3, 4E). Similarly, the cell wall remodeling enzyme D-alanine-D-alanyl carrier protein ligase (*dlt*A), which mediates D-alanylation of teichoic acids and thus resistance to AMPs (Simanski et al., 2013), was highly expressed in both anatomical niches (Figures 3, 4F). In contrast, the putative chitinase-encoding SE0760 (Zhang et al., 2003) lacked nasal expression but showed high expression on skin (Figures 3, 4G).

#### Adhesins

For the permanent colonization, factors involved in tissue adherence seem to be important. In the authentic environment, we found increased transcription of the adhesion *sdr*G at both anatomical sites and, with greater heterogeneity between individuals, for *sdr*H as well (Figures 3, 4H,I). In contrast, *ebp* encoding the elastin-binding protein was not induced under *in vivo* conditions indicating a selective regulation of adhesion molecules (Figure 3; Supplementary Figure 1).

Wall teichoic acid (WTA) of *S. aureus* is known to be an important colonization factor mediating attachment to epithelial and endothelial cells (Weidenmaier et al., 2005). In *S. epidermidis*, WTA is required for the primary attachment and accumulation phase of the *S. epidermidis* biofilm phenotype (Holland et al., 2011). Similar to *S. aureus*, a multitude of enzymes are involved in WTA biosynthesis. We have characterized the role of WTA during colonization by analyzing the expression of *tag*B. We detected high transcript levels in the nose, whereas on the skin *tag*B transcription was heterogeneous (Figure 4J).

#### Cell Wall Enzymes

Of the analyzed *sce*D, *aae*, and *atl*E genes belonging to the “cell wall enzymes” category, only autolysin *sce*D played a critical role in all individuals studied, as shown by its high transcription *in vivo* (Figures 3, 4K).

### Niche-Specific Gene Expression of *Staphylococcus epidermidis* During Commensalism

**Figure 5:**
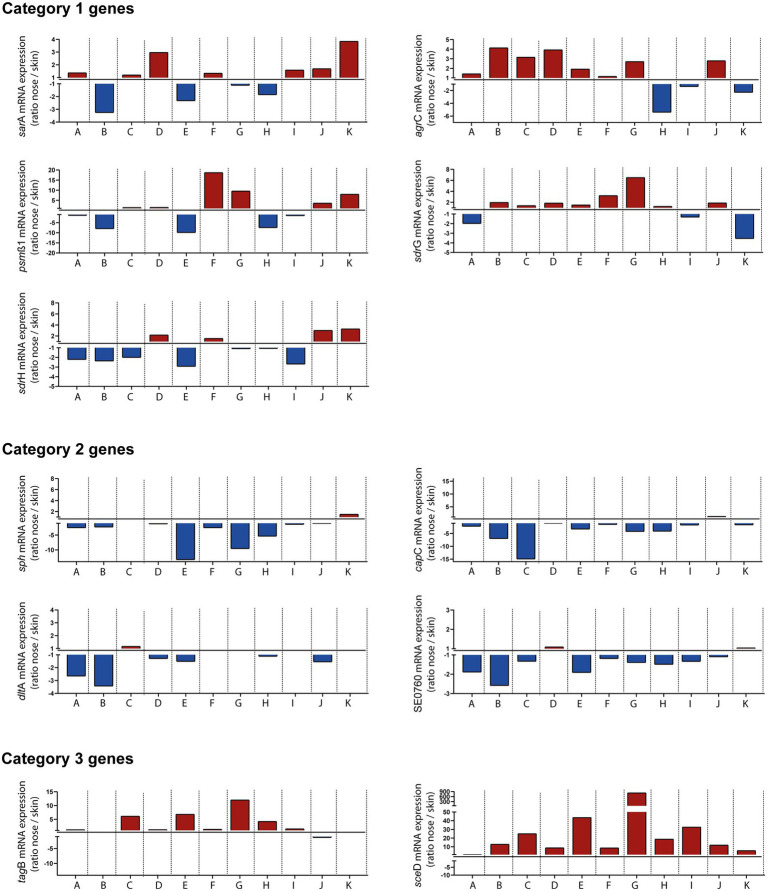
*In vivo* transcription of indicated *Staphylococcus epidermidis* genes in the nose vs. skin of 11 healthy individuals (A–K). Transcripts were quantified in reference to the constitutively expressed gene *gyr*B and the ratio of transcription between nose and skin was plotted. Red columns indicate high expression in the nose and blue columns indicate high expression on skin. Category 1 genes heterogeneously expressed between individuals. Category 2 genes increased transcription on skin. Category 3 genes increased transcription during nasal colonization. Gene name abbreviations, see Supplementary Table 2.

Collection of swab material both from nose and skin of all individuals also enabled a comparative analysis of transcript levels between both sites (without considering *in vitro* expression as a reference). This comparison revealed three distinct categories of genes: Category 1 included genes that were heterogeneously expressed between individuals and therefore sometimes showed higher transcription in the nose and sometimes on the skin, like *sar*A, *agr*, *psm*ß1, *sdr*G, and *sdr*H (Figure 5). Category 2 included genes like *sph*, *cap*C, *dlt*A, and SE0760 whose transcription was higher on the skin. Finally, category 3 contained genes such as *tag*B and *sce*D that were increased during nasal but not skin colonization (Figure 5). In summary, our results revealed site-specific gene expression of *S. epidermidis* during colonization of different host niches.

## Discussion

Mounting evidence indicates that bacterial gene expression profiles significantly differ between *in vitro* growth conditions and *in vivo* consistent with differential availability of signals involved in gene regulation (Goerke et al., 2000; Joost et al., 2009). However, there is only a limited number of studies addressing gene expression of staphylococcal species in the authentic human environment and these studies mainly focused on the invasive lifestyle (Joost et al., 2009; Le Masters et al., 2021). In *S. epidermidis* research, the reported transcriptional analyses focused on *in vivo* gene expression analysis in models of foreign material infections (Vandecasteele et al., 2003). Given the mainly commensal lifestyle of *S. epidermidis*, however, this approach does not facilitate a truly comprehensive picture of its biology. In fact, analysis of gene expression profiles of colonizing *S. epidermidis* is critical to gain insights into niche adaptation events during colonization of, for example, the skin. Furthermore, such analysis provides important clues to better understand the evolution of *S. epidermidis* within the host during the transition from commensalism to invasive disease. Therefore, we established qPCR assays that allowed us to quantitatively monitor gene transcription of more than 20 *S. epidermidis* genes *in vivo*. The assay was employed to analyze specimens obtained from two environments dominated by *S. epidermidis* colonization and significantly differing in environmental conditions. The nose being a moist and aerobic habitat (Grice and Segre, 2011), compared to the toe web, a moist microaerophilic environment with an increased temperature (Roth and James, 1988). Intriguingly, we observed a general expression signature during colonization that was significantly different from growth conditions *in vitro* and gene expression profiles of *S. epidermidis* also showed significant differences depending on the colonized body site. Thus, adaptation to the human host appears to be finely tuned and specific to the respective niche. As the skin and nose are not colonized with a single clonal *S. epidermidis* strain, but rather with different clonal lineages (Zhou et al., 2020), a limitation of this study is that we did not type the strains, and therefore, we could not determine if the nose and skin isolates were genetically identical.

To obtain a comprehensive picture of the adaptation of *S. epidermidis*, we investigated: virulence regulators, toxins, metabolic genes, adhesins, cell wall enzymes, and immune evasion genes. To gain insight into the underlying regulatory network, we analyzed five regulatory elements. While gene expression levels of *sig*B, *yyc*G, and *aps*R were subject to relevant individual heterogeneity, the staphylococcal accessory regulator A (*sar*A) was highly transcribed regardless of the colonized niche, suggesting a general role for this regulatory element during the asymptomatic lifestyle of *S. epidermidis*. So far, *sar*A is known to affect transcription of various genes as either an activator or repressor (Cue et al., 2012). For example, among the affected genes, the *ica* gene locus [encoding the polysaccharide intercellular adhesin (PIA)] is positively regulated by *sar*A (Tormo et al., 2005). While this study aimed to characterize *S. epidermidis* in its non-pathogenic state, it will be interesting to clarify in further studies whether *sar*A impacts the pathogenic lifestyle (for example, biofilm formation on medical devices) and proves to be a master regulator, or whether other regulators such as the alternative sigma factor B (*sig*B; which is also known to positively influence *ica* transcription; Handke et al., 2007) dominate.

In contrast to the global activity of *sar*A, the quorum-sensing system *agr* was active only on the skin. However, this habitat-dependent *agr* activity was heterogeneous among individuals, which might be explained by clonal diversity of colonizing *S. epidermidis* strains carrying different types of the *agr* quorum-sensing system. Olson et al. (2014) reported that cross-inhibitory interactions between the *agr* type I and II system as well as between the *agr* type I and III system exist. Because of these cross-inhibitory effects, *agr* may be highly transcribed on the skin in only 55% of individuals examined. In addition, using a porcine skin model, they demonstrated that a *S. epidermidis* wild-type strain (positive for *agr*) significantly enhanced skin colonization compared to an *agr* mutant (Olson et al., 2014). These data and the transcription observed here on the skin in some individuals suggest that *agr* contributes to skin colonization, although not as the only decisive factor in humans. Most recently, a modified *agr* typ of group III (IIIb) and a new *agr* group IV was found (Zhou et al., 2020). Moreover, Zhou et al. (2020) reported that the distribution of *agr* types is highly host dependent and that multiple *agr* types of *S. epidermidis* in a host niche suppress the expression of virulence factors to maintain homeostasis. Since there is also a cross talk between *agr* peptide pheromones produces by *S. aureus* and *S. epidermidis* (Otto et al., 2001), the *agr* activity in the presence of *S. aureus* might differ. The mechanisms leading to the partial activation on human skin and whether *agr* activity is switched on, for example, during co-colonization of the nose by *S. aureus* will be the subject of future investigations. Consistent with differential levels of *agr* transcription between individuals, the *psm*β1 peptide was also heterogeneously transcribed. Among *psm* peptides, mainly β-type peptides are produced by *S. epidermidis* and are known to play a role in biofilm formation (Le et al., 2019). Since the β-type peptide does not have a cytolytic character (Otto, 2009), its heterogeneous function during asymptomatic colonization remains to be determined.

Interestingly, the *sph* gene encoding sphingomyelinase (also annotated as *hlb* in *S. aureus*) appears to be of central importance during its commensal lifestyle, although *sph* transcription was even more pronounced during skin colonization than in the nose. Recently, it has been reported that sphingomyelinase activity, on the one hand, makes nutrients available to the bacterium (by cleaving sphingomyelin into phosphocholine and ceramide) and, on the other hand, contributes to the formation of the skin barrier through ceramide (Zheng et al., 2022). In *S. aureus*, colonization was increased more than 50-fold in a mouse colonization model compared to the strain in which *hlb* was not produced, due to integration of the prophage ΦSa3mw. Here, the toxin promotes colonization by damaging keratinocytes (Katayama et al., 2013). Since the adaptation strategy of *S. epidermidis* is strongly influenced by the physiological milieu of the colonized niche, we also addressed transcription of genes involved in metabolism, such as the tricarboxylic acid cycle (TCA). Under nutrient-rich conditions, TCA activity is suppressed as the bacterial demand for biosynthetic intermediates is supplied exogenously. Only when environmental conditions change and nutrients become growth-limiting, such as during the post-exponential growth phase, catabolism of non-preferred carbon sources such as acetate begins (Somerville and Proctor, 2009). At this stage, the enzymatic activity of the TCA cycle is required (Somerville et al., 2003; Somerville and Proctor, 2009). The low transcription of the TCA cycle genes studied together with the high *sph* expression indicates a very good nutrient supply in both anatomical niches, even on the skin, where one might suspect a rather lower nutrient supply compared to the nose.

To ensure persistent colonization, *S. epidermidis* expresses molecules involved in adhesion and evasion of the immune system. For immune evasion, *cap*C and *dlt*A are present in both host niches at high transcription rates. Production of the exopolymer PGA (encoded by the *cap* locus) is known to mediate resistance to AMPs and phagocytosis (Kocianova et al., 2005). In addition to high salt concentrations, which also lead to the induction of *cap* (Kocianova et al., 2005), AMPs are present in both habitats (Cole et al., 2002; Schauber and Gallo, 2008). Besides PGA, resistance to AMPs is mediated by the expression of *dlt*A, which leads to the d-alanylation of teichoic acids, thereby reducing the anionic charge of the bacterial surface (Simanski et al., 2013). Since the putative chitinase B (SE0760) is selectively expressed on skin and it is known to mediate skin invasion (Zhang et al., 2003), it would be interesting to investigate in further studies whether a SE0760 mutant is less able to colonize and persist on skin using, for example, our newly established human colonization model (Burian et al., 2021).

Tissue adherence by *S. epidermidis* seems to be mediated by a selective transcription of adhesive molecules. We found that WTA is highly expressed during nasal colonization, whereas *sdr*G is transcribed in both habitats. *Sdr*G is mainly responsible for tissue adherence by binding to fibrinogen (Hartford et al., 2001). Given the low abundance of fibrinogen on human skin, the high expression in toe web spaces could indicate additional, so far uncharacterized functions of s*dr*G. The high expression of WTA was also demonstrated during nasal colonization of *S. aureus* (Burian et al., 2010a,b). Again, similar to persistent colonization of the human nose (Burian et al., 2010b) and early colonization of the skin (Burian et al., 2021) by *S. aureus*, we observed a strong expression of the autolysin *sce*D. Interestingly, although *sce*D was expressed in both niches, it was clearly overrepresented in the nose. This high *sce*D transcription in both species underscore the importance of the lytic transglycosylase and may therefore be a useful candidate for developing a targeted therapy by inhibition of *sce*D. Such therapy could play a role especially in atopic dermatitis when massive colonization with *S. aureus* is responsible for the severity of the disease (Totte et al., 2016) and *S. epidermidis* also has a deleterious effect by expressing, for example, the protease *ecp*A (Cau et al., 2021).

In summary, we have elucidated here for the first time the expression pattern of *S. epidermidis* in the authentic human environment. Overall, a general expression signature was observed during asymptomatic colonization that was significantly different from growth *in vitro*, and specific gene expression patterns were associated with colonization of different host niches. Thus, *S. epidermidis* is able to specifically adapt to different niches in its human host. It will be interesting to investigate in further studies, e.g., how the expression pattern of *S. epidermidis* changes in diseased skin compared to healthy skin and to investigate transcription more comprehensively, e.g., by using RNAseq analysis. Multidimensional phenotypic and genotypic studies are also needed to fully understand the transition from commensalism to invasion. In conclusion, our results significantly improve our understanding of the complex interplay between host and microbe especially during the commensal lifestyle of the bacterium.

## Data Availability Statement

The raw data supporting the conclusions of this article will be made available by the authors, without undue reservation.

## Ethics Statement

The studies involving human participants were reviewed and approved by Ethics Committee of the Medical Faculty RWTH University of Aachen, Pauwelsstraße 30, 52074 Aachen. The patients/participants provided their written informed consent to participate in this study.

## Author Contributions

HR, AY, and MB contributed to study concept and design. PT and MB designed and performed the experiments. PT, AB, CW, MH, HR, AY, and MB wrote the manuscript. PT, AB, HR, AY, and MB analyzed the data. All authors contributed to the article and approved the submitted version.

## Funding

This work was supported by a grant from the START-Program of the Faculty of Medicine of the RWTH Aachen University and by CRC TRR 156 by the DFG (to AY).

## Conflict of Interest

The authors declare that the research was conducted in the absence of any commercial or financial relationships that could be construed as a potential conflict of interest.

## Publisher’s Note

All claims expressed in this article are solely those of the authors and do not necessarily represent those of their affiliated organizations, or those of the publisher, the editors and the reviewers. Any product that may be evaluated in this article, or claim that may be made by its manufacturer, is not guaranteed or endorsed by the publisher.

## Supplementary Material

The Supplementary Material for this article can be found online at: https://www.frontiersin.org/articles/10.3389/fmicb. 2022.896311/full#supplementary-material
