# Supplementary material for: The Staphylococcus epidermidis Transcriptional Profile During Carriage
Source: Front Microbiol. 2022 Apr 26;13:896311. doi: 10.3389/fmicb.2022.896311 (PMC9087046; doi:10.3389/fmicb.2022.896311)
Supplement: Supplementary file 1 [file Table_1.DOCX]

**Supplementary Figure 1**

Global regulators


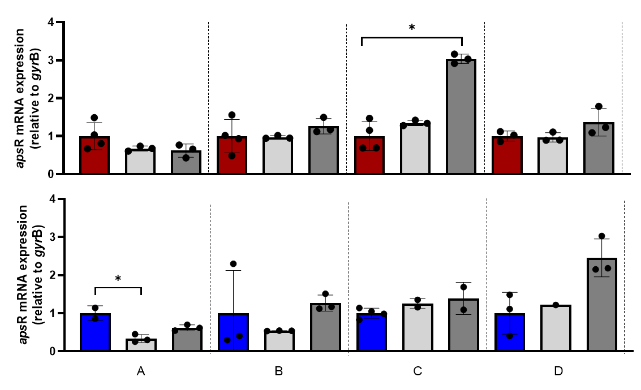

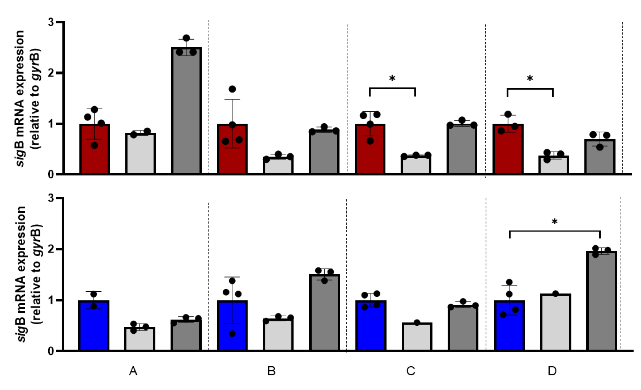

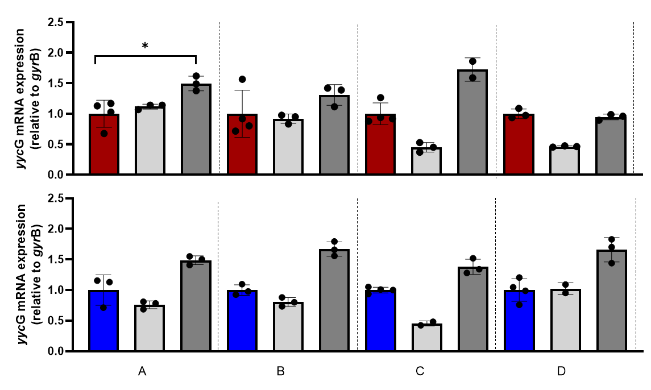


Metabolic genes


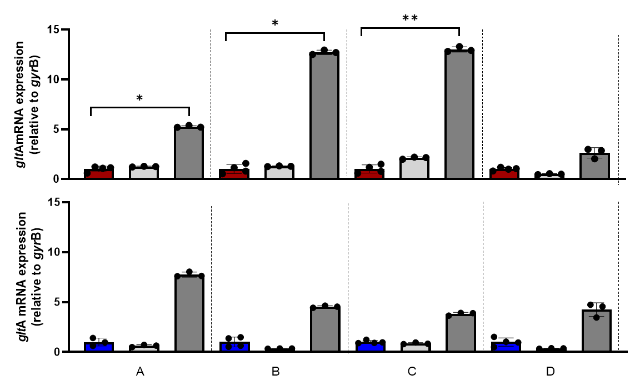

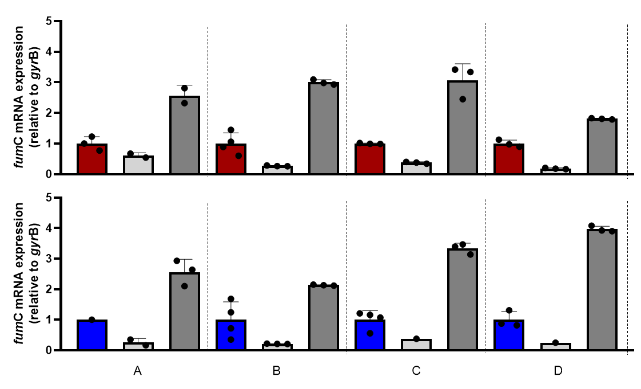

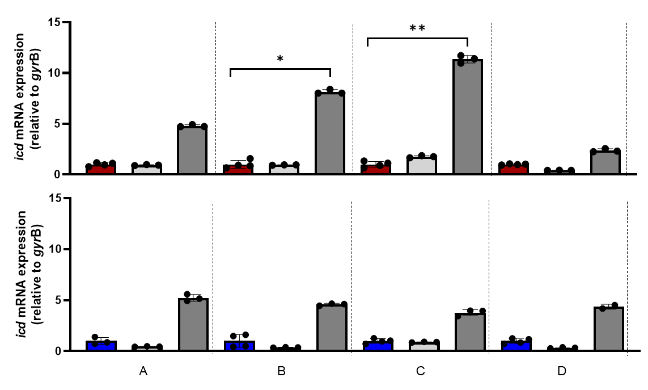


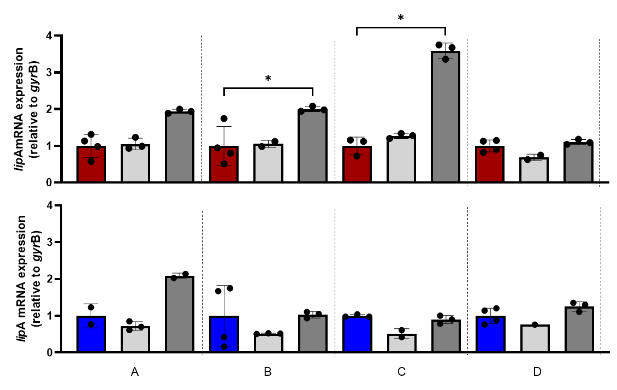


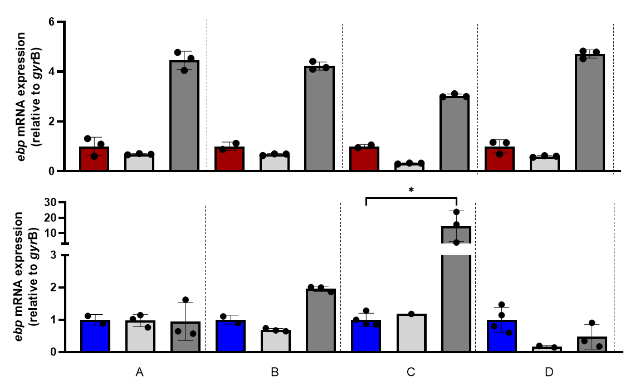

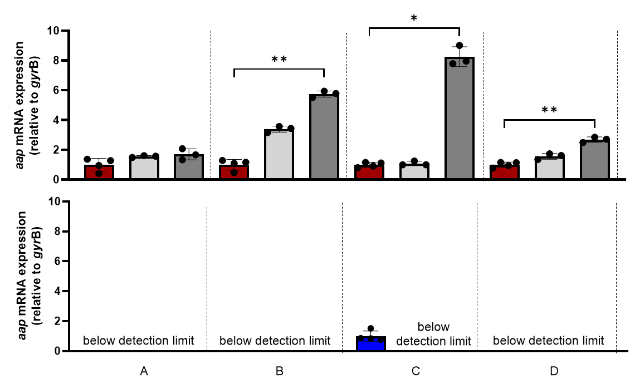
Immune evasion & colonization


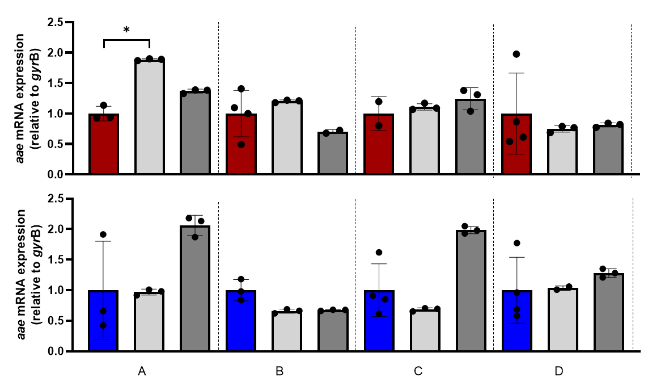

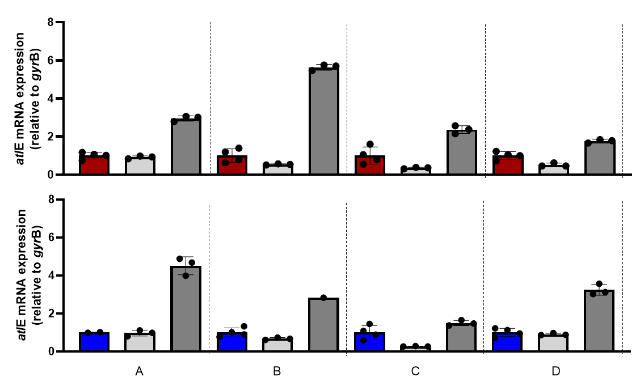
Cell wall enzymes

**Supplementary Figure 1: Transcriptional analysis of the indicated *S. epidermidis* genes in swab material of the nose and skin of four healthy individuals (A – D).** Expression levels in nose swabs (upper panels, red columns) and skin swabs (lower panels, blue columns) as well as during exponential growth *in vitro* (light grey) and post-exponential growth *in vitro* (dark grey) were normalized to the expression level of the house keeping gene *gyr*B. Values from four independent samplings (4 swabs) were used to calculate the mean expression *in vivo*. *In vitro* values represent technical replicates from one cultivation. The *in vitro* values were normalized to the respective *in vivo* level (set to 1). Statistically significant differences are indicated. *, p ≤ 0.05; **, p ≤ 0.01. Gene name abbreviations see Supplementary Table 2.
