# Supplementary material for: The Staphylococcus epidermidis Transcriptional Profile During Carriage
Source: Front Microbiol. 2022 Apr 26;13:896311. doi: 10.3389/fmicb.2022.896311 (PMC9087046; doi:10.3389/fmicb.2022.896311)
Supplement: Supplementary file 2 [file Table_2.DOCX]

Supplementary Table 1: **Oligonucleotide primers**

| **Target gene** | **Primer** | | **Primer sequence** | | **Purpose** | | |  |  |
| --- | --- | --- | --- | --- | --- | --- | --- | --- | --- |
| *gyr*B | | gyrB_F_epi | CTCGAAGCGGTTCGTAAAAG | | | qPCR | | |  |
|  | | gyrB_R_epi | TACCACGGCCATTGTCAGTA | | | qPCR | | |  |
| *agr*C | | agrC_L | TCATGAGTTGTTTGAACAAGGTTT | | | qPCR | | |  |
|  | | agrC_R | CAAAGTAACCATTTTCGATGACAG | | | qPCR | | |  |
| *sig*B | | sigB_L | TGAAATCGCACAACGCTTAG | | | qPCR | | |  |
|  | | sigB_R | TCCACACTCAGGGCATTGTA | | | qPCR | | |  |
| *sar*A | | sarA_L | AATCAGCTTTGAAGAATTTGCAG | | | qPCR | | |  |
|  | | sarA_R | TTGAGGTTGTTTATAATTTAAGTGATTGAT | | | qPCR | | |  |
| *yy*cG | | yycG_L | GTCACACGCGAAGAGACTGA | | | qPCR | | |  |
|  | | yycG_R | GATGGTTTGTTGCGGAATTT | | | qPCR | | |  |
| *aps*R | | apsR_L | GACGCGTTTATGAATTTGGAG | | | qPCR | | |  |
|  | | apsR_R | AAATCCACAGTAGCGTCTTGC | | | qPCR | | |  |
| *psm*β1 | | *psm*β1_L | AGAAGCAGCCATCACTAACG | | | qPCR | | |  |
|  | | *psm*β1_R | TTCCCAAAAATCGATTCACC | | | qPCR | | |  |
| *sph* | | sph_L | TAAATGGCCTATCGTACAACAAGA | | | qPCR | | |  |
|  | | sph_R | ATCTTTTCCTTTAAAGCATGTTGG | | | qPCR | | |  |
| *fum*C | | fumC_L | AATGTGAGCAATTACTATCAGAATCAA | | | qPCR | | |  |
|  | | fumC_R | TGAGCATTAATTCCCGTTCC | | | qPCR | | |  |
| *glt*A | | gltA_L | ACACCAATATTTGCAGTAAGTCGT | | | qPCR | | |  |
|  | | gltA_R | GGTCGCATAATTCTATTGTCTCG | | | qPCR | | |  |
| *icd* | | icd_L | GCCACAAGAAACGTTAGAAACA | | | qPCR | | |  |
|  | | icd_R | TTGTCTCAGTGCTACGTTTAATGA | | | qPCR | | |  |
| *lip*A | | lipA_L | GCGATTATGAAGCCCTTGAA | | | qPCR | | |  |
|  | | lipA_R | TCGTTTCACCTAACCCAACC | | | qPCR | | |  |
| *aap* | | aap_L | ACCTACAACTTCAGAACCTGTGAAT | | | qPCR | | |  |
|  | | aap_R | TAACCGTAGTTGGCGGTATATCT | | | qPCR | | |  |
| *cap*C | | capC_L | TATTTGCTGAGAAATTTGGGATT | | | qPCR | | |  |
|  | | capC_R | CAAAGCTAAATAACCTGGAACGA | | | qPCR | | |  |
| *sdr*G | | sdrG_L | AAACTGTGACAATGCAAACGA | | | qPCR | | |  |
|  | | sdrG_R | GACCTGAACTTGTAGAGAAAGCAAT | | | qPCR | | |  |
| *sdr*H | | sdrH_L | TGAAAATTTAGATGATCAGGTTTTAGC | | | qPCR | | |  |
|  | | sdrH_R | TGATTTTTATCTTCTTTATCCGTAGGT | | | qPCR | | |  |
| *ebp* | | ebp_L | CAGGCAACTCAAGGTCAACA | | | qPCR | | |  |
|  | | ebp_R | GCTTAATCCATTCGCACGTT | | | qPCR | | |  |
| *tag*B | | tagB_L | TGAAACCTTTGTTCCTGGTAAAAT | | | qPCR | | |  |
|  | | tagB_R | AACGGCTTCACCTTTTCTAATTC | | | qPCR | | |  |
| *sce*D | | sceD_L | TGCTCCAAGTCAATATAAAGGTGT | | | qPCR | | |  |
|  | | sceD_R | TTTACTGCTGCTCGGTCTTG | | | qPCR | | |  |
| *aae* | | aae_L | TTGGAACAACTGCCCTTAGC | | | qPCR | | |  |
|  | | aae_R | TATGCGTTGTTGCAGCTTG | | | qPCR | | |  |
| *atl*E | | atlE_L | GATGGCGAGATTGCTTTCA | | | qPCR | | |  |
|  | | atlE_R | TCTGTCGGAGCTGTTTCTATAATTC | | | qPCR | | |  |
| *dlt*A | | dltA_L | GAAGCAGTTGCGGTTAGACA | | | qPCR | | |  |
|  | | dltA_R | TGATTCTTCATCAAGTTGCTTATATGT | | | qPCR | | |  |
| SE0760 | | SE0760_L | AAAGGCATAAGTGCTTACGATAAAG | | | qPCR | | |  |
|  | | SE0760_R | GCTTTGGATGTATCAACTTGACC | | | qPCR | | |  |
|  |  | | |  | | |  | | |
