# Supplementary material for: The Staphylococcus epidermidis Transcriptional Profile During Carriage
Source: Front Microbiol. 2022 Apr 26;13:896311. doi: 10.3389/fmicb.2022.896311 (PMC9087046; doi:10.3389/fmicb.2022.896311)
Supplement: Supplementary file 3 [file Table_3.DOCX]

Supplementary Table 2: ***S. epidermidis* genes analyzed by qPCR**

| **Product** | **Gene** | **Function** |
| --- | --- | --- |
| **Virulence regulators** |  |  |
| Staphylococcal accessory regulator A | *sar*A | Virulence regulator |
| Accessory gene regulator | *agr*C | Virulence regulator |
| Regulator of cell wall metabolism and virulence | *yyc*G | Virulence regulator |
| Alternative sigma factor B | *sig*B | Virulence regulator |
| Antimicrobial peptide-sensing system | *aps*R | Virulence regulator |
| **Toxins** |  |  |
| Sphingomyelinase | *sph* | Contributes to colonization and skin barrier homeostasis |
| Phenol-soluble modulin | *psm*β1 | Biofilm structuring and dispersal |
| **Metabolic genes** |  |  |
| Fumarase | *fum*C | TCA cycle enzyme (catalyzes the conversion of fumarate to L-malate) |
| Citrate synthasec | *glt*A | TCA cycle enzyme (catalyzes the conversion of oxaloacetate and acetyl-coenzyme A into citrate and coenzyme A) |
| Isocitrate dehydrogenase | *icd* | TCA cycle enzyme (catalyzes the oxidation and decarboxylation of isocitrate to α-ketoglutarate) |
| Lipase A | *lip*A | Possible degrade lipids |
| **Immune evasion & colonization** |  |  |
| Accumulation-associated protein | *aap* | Biofilm formation |
| Poly-γ-glutamic acid (PGA) | *cap*C | Resistance to AMPs and phagocytosis |
| D-alanine-D-alanyl carrier protein ligase | *dlt*A | Resistance to AMPs by d-alanylation of teichoic acids |
| Chitinase B | SE0760 | Invasion of skin |
| **Adhesins** |  |  |
| Fibrinogen binding protein | *sdr*G | Binds to fibrinogen |
| Serine-aspartic acid rich cell surface adhesin | *sdr*H | Not known |
| Elastin-binding protein | *ebp* | Binds to elastin |
| Wall teichoic acid | *tag*B | Bacterial adhesion |
| **Cell wall enzymes** |  |  |
| *Staphylococcus carnosus* exoprotein D | *sce*D | Lytic transglycosylase |
| Autolysin | *aae* | Bifunctional autolysin and adhesin |
| Autolysin | *atl*E | Bifunctional autolysin and adhesin |
